# Improving the Accuracy and Speed of Visual Field Testing in Glaucoma With Structural Information and Deep Learning

**DOI:** 10.1167/tvst.12.10.10

**Published:** 2023-10-13

**Authors:** Giovanni Montesano, Georgios Lazaridis, Giovanni Ometto, David P. Crabb, David F. Garway-Heath

**Affiliations:** 1 City, University of London, Optometry and Visual Sciences, London, UK; 2 NIHR Biomedical Research Centre, Moorfields Eye Hospital NHS Foundation Trust and UCL Institute of Ophthalmology, London, UK; 3 Centre for Medical Image Computing, University College London, London, UK

**Keywords:** automated perimetry, artificial intelligence, optical coherence tomography, glaucoma, structure–function

## Abstract

**Purpose:**

To assess the performance of a perimetric strategy using structure–function predictions from a deep learning (DL) model.

**Methods:**

Visual field test–retest data from 146 eyes (75 patients) with glaucoma with (median [5th–95th percentile]) 10 [7, 10] tests per eye were used. Structure–function predictions were generated with a previously described DL model using cicumpapillary optical coherence tomography (OCT) scans. Structurally informed prior distributions were built grouping the observed measured sensitivities for each predicted value and recalculated for each subject with a leave-one-out approach. A zippy estimation by sequential testing (ZEST) strategy was used for the simulations (1000 per eye). Ground-truth sensitivities for each eye were the medians of the test–retest values. Two variations of ZEST were compared in terms of speed (average total number of presentations [NP] per eye) and accuracy (average mean absolute error [MAE] per eye), using either a combination of normal and abnormal thresholds (ZEST) or the calculated structural distributions (S-ZEST) as prior information. Two additional versions of these strategies employing spatial correlations were tested.

**Results:**

S-ZEST was significantly faster, with a mean average NP of 213.87 (SD = 28.18), than ZEST, with a mean average NP of 255.65 (SD = 50.27) (*P* < 0.001). The average MAE was smaller for S-ZEST (1.98; SD = 2.37) than ZEST (2.43; SD = 2.69) (*P* < 0.001). Spatial correlations further improved both strategies (*P* < 0.001), but the differences between ZEST and S-ZEST remained significant (*P* < 0.001).

**Conclusions:**

DL structure–function predictions can significantly improve perimetric tests.

**Translational Relevance:**

DL structure–function predictions from clinically available OCT scans can improve perimetry in glaucoma patients.

## Introduction

Visual field (VF) testing, or standard automated perimetry (SAP), is a staple of clinical care in glaucoma and the most important test for diagnosis and monitoring of progression. SAP is usually performed by asking patients to fixate a central target while round light stimuli of varying intensity are projected at different locations on their retina. The patient responds by pressing a button when a stimulus is perceived. The responses are then elaborated by the machine to produce retinal sensitivity maps. By its nature, the test requires strong cooperation from tested subjects, who need to provide timely and accurate responses and maintain central fixation throughout the test. Despite multiple efforts to improve the test, SAP is still fraught with substantial test–retest variability^1^ and can be taxing for the patient.^2^ These problems are usually amplified in patients with advanced VF damage.^1^^,^^3^

Imaging of the retina and of the optic nerve head (ONH) has become a prominent aspect of clinical care, especially with the introduction of optical coherence tomography (OCT). OCT provides tomographic imaging of the retinal tissue, allowing qualitative assessment and quantitative measurement of different retinal layers. Glaucoma care is specifically concerned with evaluation of the retinal ganglion cell (RGC) layer and the retinal nerve fiber layer (RNFL), where RGC axons reside. A loss of RGCs and their axons manifests as thinning of the RNFL and corresponding VF sensitivity loss. However, modeling the relationship between RNFL damage and VF loss, especially in more advanced glaucoma, has proven challenging because of its particular spatial features, its nonlinear nature,^4^ and the high interindividual variability.^5^^–^^8^ In recent years, artificial intelligence (AI) and deep learning (DL) methods, especially those based on convolutional neural networks (CNNs), have significantly improved the prediction of SAP sensitivity from OCT data,^9^^–^^14^ with some demonstrating impressive accuracy.^9^

Although a full replacement of SAP with OCT derived metrics is unlikely, structural information can be incorporated into the VF test to improve accuracy and speed.^15^^,^^16^ Improvements are expected to be greater the closer the structural prediction is to the true underlying VF sensitivity.^17^ The application of AI for this purpose is appealing. The objective of our study was to use a DL algorithm, recently developed by our group, to predict SAP sensitivity from both segmented peripapillary RNFL thickness profiles and peripapillary OCT images, with the aim to improve the speed and accuracy of VF tests. We performed simulation experiments with an open-source freely available platform, the Open Perimetry Interface (OPI),^18^ to evaluate the improvement brought by the integration of structural DL predictions as prior information into a Bayesian testing strategy. The data used for the simulations were derived from a cohort of patients with glaucoma who underwent multiple VF testing and OCT imaging sessions,^19^ allowing accurate assessment of the performance of the proposed structure-based strategy. We specifically evaluated the improvement in the speed of the test and the accuracy of the estimation of both pointwise sensitivity and global metrics.

## Methods

### Visual Field and Imaging Data

#### Compass Validation Study

Four hundred forty-four healthy and 499 glaucoma subjects were recruited to a study designed to compare the clinical performance of the Humphrey Field Analyzer (HFA; Carl Zeiss Meditec, Dublin, CA) and the Compass perimeter (CenterVue, Padova, Italy).^20^ Only data obtained from the HFA were used in this research and are described in the following. The tests were performed at eight study sites, with five sites acquiring OCT images with the spectralis (Heidelberg Engineering, Heidelberg, Germany). The study was undertaken in accordance with good clinical practice guidelines and adhered to the tenets of the Declaration of Helsinki. All patients gave their written informed consent to participate in the study. Ethics committee approval was obtained (International Ethics Committee of Milan, Zone A, 22/07/2015, ref: Prot. no. 0019459), and the study was registered as a clinical trial (ISRCTN13800424). Details can be found in the original paper.^9^ Briefly, healthy subjects were required to have a normal optic nerve head in both eyes, intraocular pressure less than 21 mmHg in both eyes, and no other signs of ocular disease. Glaucoma subjects were required to have glaucomatous optic neuropathy (GON), defined as glaucomatous changes to the ONH or RNFL as determined by a specialist from fundus photographs or spectral-domain optical coherence tomography (SD-OCT), independently of the VF; to be receiving anti-glaucoma therapy; and to have no ocular pathologies, other than glaucoma, in the tested eyes. All subjects performed a perimetric test with the HFA 24-2 grid (SITA Standard) to both eyes (if both eligible). Fundus pictures with the COMPASS perimeter and SD-OCT scans of the ONH and the circumpapillary RNFL were acquired for the purpose of clinical confirmation of GON; the acquisition of OCT data was not subject to a standardized procedure. As explained in the original paper,^9^ the training of the DL algorithm used for this study (see later) included only eyes with a circumpapillary RNFL scan performed with a SPECTRALIS SD-OCT (954 eyes from 552 people, 332 with GON). All data from healthy eyes were used to build the normative prior (see later). Descriptive statistics of this dataset have been extensively reported elsewhere.^9^^,^^20^

#### RAPID Study

Eighty-two clinically stable glaucoma patients were recruited to a test–retest study.^19^ The study was undertaken in accordance with good clinical practice guidelines and adhered to the tenets of the Declaration of Helsinki. The study was approved by the North of Scotland National Research Ethics Service committee (reference no. 13/NS/0132), and NHS Permissions for Research were granted by the Joint Research Office at University College London Hospitals NHS Foundation Trust on December 3, 2013. All patients provided written informed consent before the screening investigations were carried out. Patients were required to have reproducible VF loss with corresponding damage to the ONH and no other condition that could lead to VF loss; to be > 18 years old; and to have a visual acuity of at least 20/40, a refractive error within ±8 diopters (D), and an intraocular pressure of < 30 mmHg. The VF mean deviation (MD) had to be better than −16 decibels (dB) in the worse eye and better than −12 dB in the better eye. VF testing and OCT imaging were carried out twice at the first visit and once at each subsequent visit, up to 10 times within a 3-month period. VF testing was undertaken with an HFA, and circumpapillary OCT imaging was carried out using a SPECTRALIS SD-OCT (software version 5.2.4). The final dataset was composed of 1396 test repeats performed in 146 eyes of 75 subjects. The median number of test repeats per eye was 10 (5th, 95th percentile: 7, 10), with a minimum of three for inclusion. Only nine eyes had fewer than nine tests. All tests had a percentage of false-positive errors ≤ 15% (only one test reached this threshold; median: 1; 5th, 95th percentile: 0, 7). Baseline characteristics of the selected RAPID study test–retest cohort are reported in Table 1.

**Table 1.** Baseline Characteristics of the RAPID Test–Retest Cohort

Characteristic	Median [Interquartile Range]
Age (y)	70 [64, 76]
BCVA (logMAR)	0 [−0.08, 0.18]
SE (D)	0 [−1.35, 0.88]
IOP (mmHg)	14 [12, 16]
Average MD (dB)	−3.29 [−7.76, −1.24]
Average PSD (dB)	4.26 [2.16, 9.60]
Average VFI (%)	94 [78, 98]
Average cp-RNFLT (µm)	71.7 [59.6, 84.9]

Cohort included 80 females and 66 males. BCVA, best-corrected visual acuity; SE, spherical equivalent; IOP, intraocular pressure; MD, mean deviation; PSD, pattern standard deviation; VFI, visual field index; cp-RNFLT, circumpapillary retinal nerve fiber layer thickness.

### DL Structural Prediction of Sensitivity

**Figure 1.**
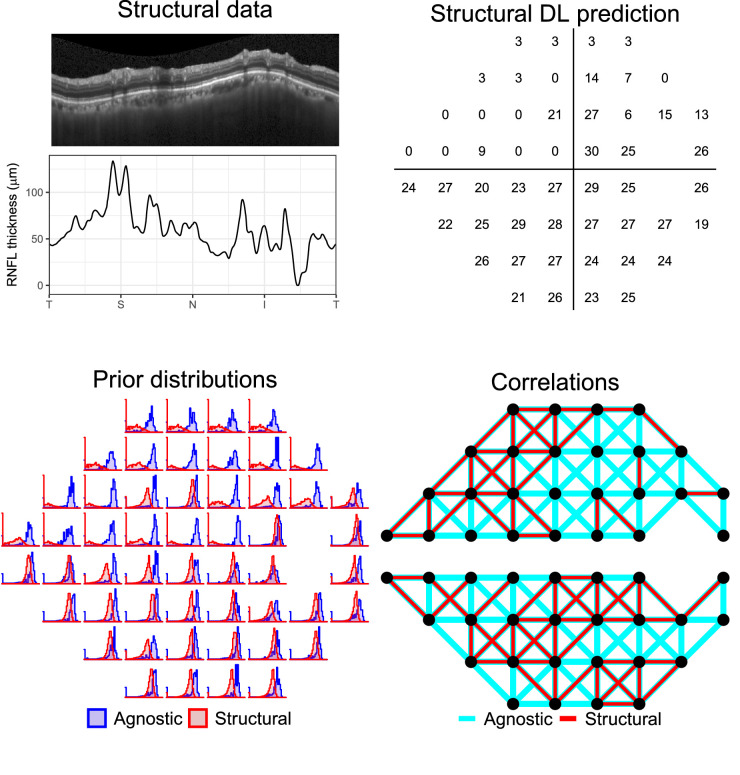
Example of a DL prediction from structural data (*top panels*). The *bottom panels* show the prior distributions and spatial correlation structures used by ZEST (agnostic) and S-ZEST (structural).

The DL algorithm used for this work has been previously presented in detail.^9^ Briefly, the method uses a stacked generalization ensemble technique to combine the results of two submodels. The first submodel is a multi-input CNN and the second submodel is a multichannel variational autoencoder. Both submodels take the SD-OCT B-scan image and the corresponding segmented RNFL thickness profile as inputs. The predictions from the two models are then combined through a third architecture, XGBoost,^21^ which generates the ensemble prediction. An example of the prediction process is shown in Figure 1. A VF prediction was generated for each of the OCT scans available in the RAPID dataset, so that each eye could have multiple structurally predicted VFs.

### Simulation Experiments

#### Implementation of the Perimetric Strategies

We used the OPI platform^18^ for R (R Foundation for Statistical Computing, Vienna, Austria) to implement different versions of the zippy estimation by sequential testing (ZEST) strategy.^22^ This is a Bayesian strategy that determines sensitivity at a specific location by progressively updating a starting prior distribution of possible outcome values (usually from 0 to 40 dB) with responses from the observer to generate a posterior distribution. The updating is performed through a likelihood function which often has the shape of the cumulative distribution function of a Gaussian distribution with a standard deviation (SD) of 1 dB and asymptotes at 0.03 and 0.97 and is centered on the intensity of the current presentation. At each step, the presentation is chosen as the mean of the updated (posterior) distribution, which becomes the prior distribution for the next step in the strategy. The algorithm can be stopped by applying various criteria. For our implementation, the stopping criterion was a SD of the posterior distribution ≤ 1.5 dB^23^ or when a maximum of 10 presentations was reached.

An optimal choice of prior distributions can make the test faster, more precise, and generally more efficient. For most implementations, the prior distribution for each location is built as a mixture of normal and abnormal sensitivity values weighted 4:1,^24^ and this was the choice for our implementation of the standard ZEST. For consistency with the training process of the DL algorithm, the distribution of normal values for each of the 52 VF locations was derived from the HFA tests from the 444 healthy participants in the COMPASS validation study.^20^ The distribution of abnormal locations was derived from Turpin et al.^24^ Alternatively, bespoke prior distributions can be created for each location using structural information. For our experiments, the structure-derived prior distributions were built by grouping all the observed sensitivity values in the RAPID dataset for each (rounded) sensitivity value predicted by the DL algorithm. To avoid biasing our results, the prior distributions for each subject were built by excluding both eyes from that subject from the sample (leave-one-out). For the structure-informed strategy, an appropriate structure-derived prior was then chosen for each location based on the sensitivity predicted from the OCT data by the DL algorithm. We call this algorithm structural ZEST (S-ZEST). We finally tested two additional versions of the strategies that employed spatial correlations to improve speed. We refer to this process as spatial enhancement. Following Rubinstein et al.,^25^ nearest neighbor locations were connected, but the connections were not allowed to cross the horizontal midline. Responses that were provided for one location also updated the prior distribution of its nearest neighbors with a scaled likelihood. For the standard ZEST, the scaling factor was 0.2,^25^ meaning that the evidence provided by the response to one location to update the neighbors was less than that for the tested location itself. For the structural strategy, nearest neighbors were also disconnected if their sensitivities predicted from structure were more than 3 dB apart. This was meant to use structural information to preserve the edges of the scotomas. This also provided stronger confidence in the similarity between connected nearest neighbors. Therefore, for S-ZEST, the scaling coefficient was set to 0.4. An example application of the two strategies is shown in Figure 1. For all of our simulations, all locations that had not reached the termination criteria were tested randomly. Some improvement could be obtained by implementing growth patterns or by prioritizing locations with larger variance in their prior distribution.^25^

#### Perimetric Responses

Perimetric responses were simulated using a Gaussian psychometric function with 5% false-positive (FP) and 5% false-negative (FN) errors. The equation for the psychometric function was

$$\begin{array}{ccc} & & \;P=FP+\left(1-FP-FN\right)\\ & & \;\times \left[1-\Phi \left(\mathrm{dB},\mathrm{True}\;\mathrm{sensitivity},\mathrm{SD}\right)\right] \end{array}$$

where *P* indicates the probability of response to a certain stimulus intensity (dB in the equation), and Φ indicates the Gaussian cumulative distribution function. The simulations were also performed with high FPs (20%) and high FNs (20%). The true sensitivity was set as the best available estimate (BAE) from the RAPID dataset for each location in each eye (i.e., the median of all the observed test–retest sensitivity values). The slope of the psychometric function (i.e., the SD of the Gaussian) was changed with the true sensitivity according to the equation reported by Henson et al.^3^ for a mixture of healthy and glaucomatous patients to simulate the change in variability with glaucomatous damage. The SD was capped at a maximum of 6 dB. The OPI package for R was used for all the simulations. However, because one of the goals was to evaluate the effect of incorporating structural information on global metrics, we modified our simulation procedure to incorporate correlations in the responses among different locations within the same test. This is important, because simulating completely uncorrelated errors at each location can lead to a severe underestimation of the global fluctuations in the VF, which might artificially reduce the variability of the global metrics, such as mean sensitivity (MS) or MD.^26^^–^^28^ This phenomenon is also known as the global visit effect (GVE).^26^ The details of how the GVE was incorporated in the simulations are provided in the Appendix, largely based on the work by Wu and Medeiros.^27^ For S-ZEST, multiple structurally predicted VFs were available, one from each of the repeated OCT scans. To replicate clinical practice, the prediction from a random OCT scan was chosen from those available for a given eye at each simulated test.

### Evaluation of the Results

The results were quantified by measuring the mean absolute error (MAE) for both pointwise sensitivity and MS for the different strategies. Speed was quantified by measuring the number of presentations required to determine each threshold and for each 24–2 test. The MAE and average total number of presentations per test were also calculated for each eye. Because in simulation experiments the sample size can be arbitrarily decided by the researcher by increasing the number of simulations, we only tested significance for the metrics calculated for each eye—that is, error in MS, MAE per eye, and average total number of presentations per eye (*N* = 146 eyes). Note that the estimate of the average MAE, but not its variability, is the same regardless of how it is calculated. The different strategies were compared by reliability score (FN and FP) using linear mixed models, to account for results obtained from the same eye. All calculations were performed in R (R Foundation for Statistical Computing, Vienna) using the *lme4* package.^29^

## Results

### Accuracy of the Perimetric Strategies

**Figure 2.**
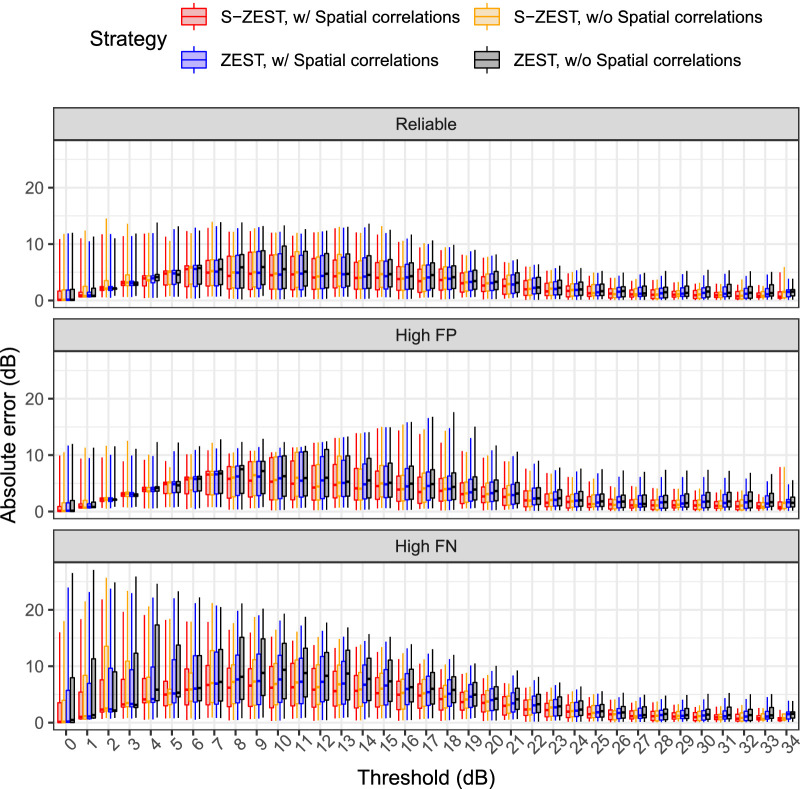
Box plots of the absolute error for pointwise thresholds for reliable observers (5% FPs and FNs), high FPs (20%), and high FNs (20%). The *horizontal line* indicates the median, the *boxes* include the interquartile range, and the *whiskers* extend to the 5th and 95th percentiles.

**Figure 3.**
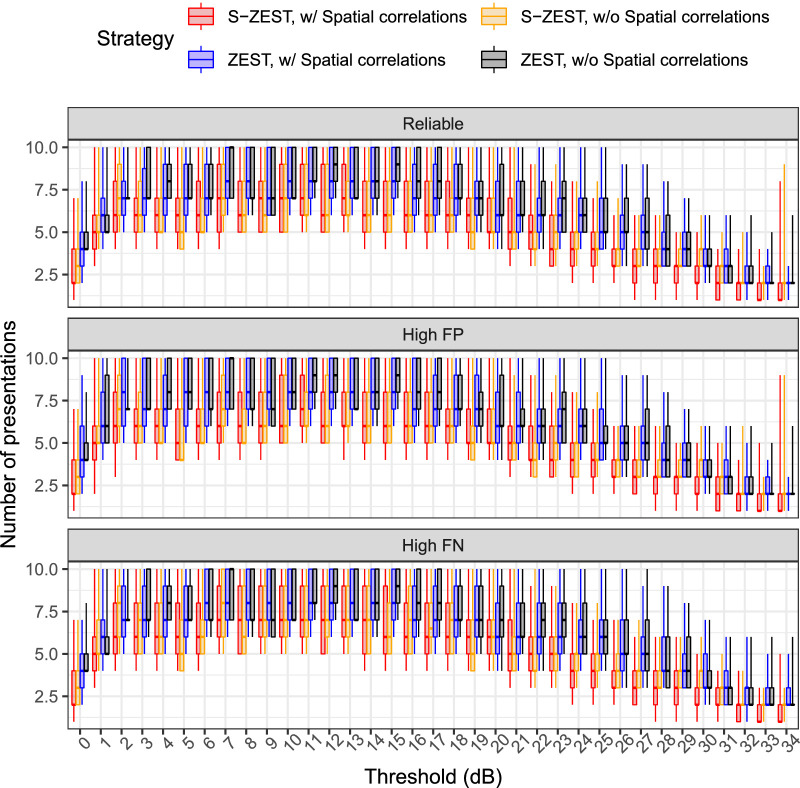
Box plots of the number of presentations required for the determination of pointwise thresholds for reliable observers (5% FPs and FNs), high FPs (20%), and high FNs (20%). The *horizontal line* indicates the median, the *boxes* include the interquartile range, and the *whiskers* extend to the 5th and 95th percentiles.

The box plots in Figure 2 report the pointwise absolute error (AE) at each sensitivity for the different strategies at three levels of reliability. The box plots in Figure 3 report the pointwise number of presentations required to determine the threshold at each sensitivity for the different strategies at three levels of reliability. These results are summarized in Table 2.

**Table 2.** Pointwise Average (SD) for the Mean Signed Error, Mean Absolute Error, and Number of Presentations for Reliable Observers, High FPs (20%), and High FNs (20%)

		Mean Signed Error		Mean Absolute Error		Average Presentations	
	Reliability	ZEST	S-ZEST	ZEST	S-ZEST	ZEST	S-ZEST
Without spatial correlations	Reliable	0.27 (3.62)	−0.23 (3.08)	2.43 (2.69)	1.98 (2.37)	4.92 (2.32)	4.11 (1.81)
With spatial correlations		0.17 (3.28)	−0.12 (2.91)	2.18 (2.45)	1.83 (2.27)	4.75 (2.20)	3.56 (2.07)
Without spatial correlations	High FPs	1.40 (4.57)	0.43 (3.39)	2.97 (3.75)	2.13 (2.68)	4.63 (2.31)	4.01 (1.78)
With spatial correlations		1.23 (3.87)	0.62 (3.16)	2.61 (3.12)	2.00 (2.52)	4.56 (2.25)	3.45 (2.04)
Without spatial correlations	High FNs	−1.32 (5.24)	−1.21 (3.85)	3.36 (4.24)	2.50 (3.17)	5.42 (2.42)	4.39 (1.96)
With spatial correlations		−1.22 (4.47)	−1.09 (3.47)	2.86 (3.64)	2.25 (2.85)	5.23 (2.31)	3.85 (2.18)

**Figure 4.**
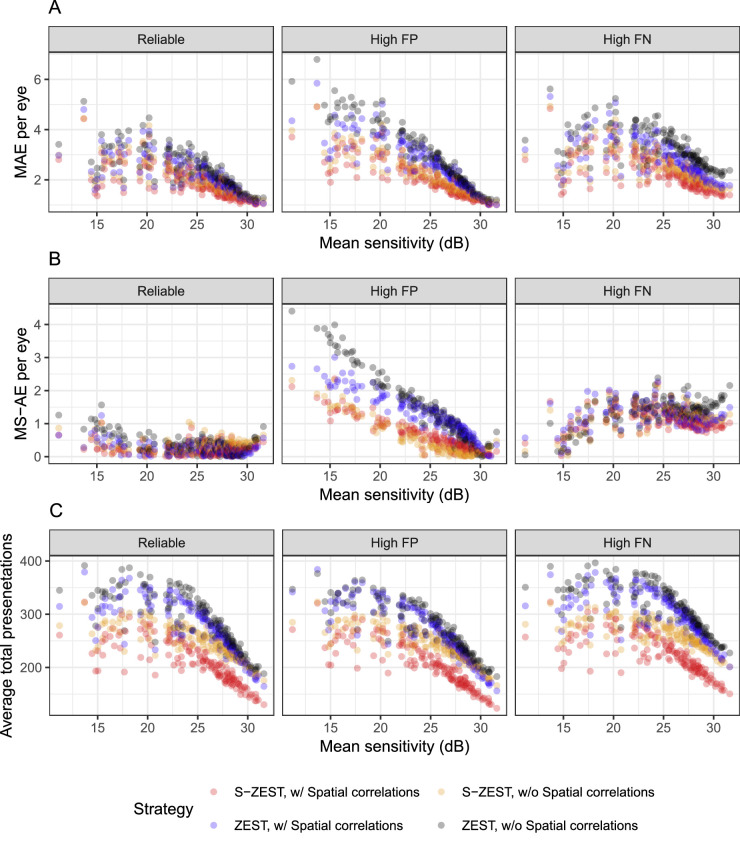
(**A**) Mean absolute error (MAE) per eye (*N* = 146) for each tested strategy, plotted by “true” assumed mean sensitivity, for reliable observers (5% FPs and FNs), high FPs (20%), and high FNs (20%). (**B**) Mean sensitivity absolute error (MS-AE), calculated as the average of the absolute difference between the estimated MS and the true MS; this metric is affected by global fluctuations in sensitivity. (**C**) Average total number of presentations required to complete the test with the different strategies.

Figure 4 reports the MAEs for the whole VF of each eye (Fig. 4A) and the AE for the MS (Fig. 4B). In general, the MAE for both implementations of S-ZEST was better than for ZEST (*P* < 0.001). The strategies employing spatial correlations performed better (*P* < 0.001). However, the difference in the mean sensitivity absolute error (MS-AE) was only significant for the observers with high FPs. Note that the variability of the MS was more strongly affected by global fluctuations. Figure 4C reports the average total number of presentations for the whole VF test of each eye. In general, S-ZEST greatly improved speed, with a marginal additional contribution from the use of spatial correlations, especially at higher thresholds (*P* < 0.001). These results are summarized in Table 3. *P* values for all pairwise comparisons are reported in Table 4.

**Table 3.** Average (SD) Mean Absolute Error (MAE) and Mean Sensitivity Absolute Error (MS-AE) Per Eye (*N* = 146) for Each Tested Strategy for Reliable Observers (5% FPs and FNs), High FPs (20%), and High FNs (20%)

		MAE Per Eye		MS-AE Per Eye		Total Presentations Per Test	
	Reliability	ZEST	S-ZEST	ZEST	S-ZEST	ZEST	S-ZEST
Without spatial correlations	Reliable	2.43 (0.77)	1.98 (0.61)	0.38 (0.30)	0.34 (0.19)	255.65 (50.27)	213.87 (28.18)
With spatial correlations		2.18 (0.70)	1.83 (0.61)	0.25 (0.20)	0.22 (0.17)	247.06 (47.28)	185.18 (37.13)
Without spatial correlations	High FPs	2.97 (1.23)	2.13 (0.77)	1.44 (1.01)	0.50 (0.54)	240.61 (47.18)	208.40 (29.19)
With spatial correlations		2.61 (0.97)	2.00 (0.74)	1.23 (0.65)	0.62 (0.47)	236.97 (49.33)	179.18 (38.03)
Without spatial correlations	High FNs	3.36 (0.73)	2.50 (0.63)	1.33 (0.39)	1.22 (0.34)	281.93 (41.81)	228.19 (24.58)
With spatial correlations		2.86 (0.73)	2.25 (0.65)	1.22 (0.33)	1.09 (0.33)	271.95 (41.18)	200.24 (34.28)

Note that the average MAE is the same as in Table 2, but in this table its SD is determined by the variation across different eyes rather than individual simulation results for each location.

**Table 4.** Significance of All Pairwise Differences Between Strategies in Terms of Mean Absolute Error (MAE), Mean Sensitivity Absolute Error (MS-AE), and Total Number of Presentations Per Test

			*P*		
Reliability	Comparison		MAE	MS-AE	Total Presentations
Reliable	S-ZEST, with spatial correlations	S-ZEST, without spatial correlations	<0.001	0.052	<0.001
	S-ZEST, with spatial correlations	ZEST, with spatial correlations	<0.001	0.894	<0.001
	S-ZEST, with spatial correlations	ZEST, without spatial correlations	<0.001	0.006	<0.001
	S-ZEST, without spatial correlations	ZEST, with spatial correlations	<0.001	0.250	<0.001
	S-ZEST, without spatial correlations	ZEST, without spatial correlations	<0.001	0.882	<0.001
	ZEST, with spatial correlations	ZEST, without spatial correlations	<0.001	0.048	<0.001
High FPs	S-ZEST, with spatial correlations	S-ZEST, without spatial correlations	<0.001	0.077	<0.001
	S-ZEST, with spatial correlations	ZEST, with spatial correlations	<0.001	<0.001	<0.001
	S-ZEST, with spatial correlations	ZEST, without spatial correlations	<0.001	<0.001	<0.001
	S-ZEST, without spatial correlations	ZEST, with spatial correlations	<0.001	<0.001	<0.001
	S-ZEST, without spatial correlations	ZEST, without spatial correlations	<0.001	<0.001	<0.001
	ZEST, with spatial correlations	ZEST, without spatial correlations	<0.001	<0.001	0.0280
High FNs	S-ZEST, with spatial correlations	S-ZEST, without spatial correlations	<0.001	0.07	<0.001
	S-ZEST, with spatial correlations	ZEST, with spatial correlations	<0.001	0.066	<0.001
	S-ZEST, with spatial correlations	ZEST, without spatial correlations	<0.001	<0.001	<0.001
	S-ZEST, without spatial correlations	ZEST, with spatial correlations	<0.001	0.999	<0.001
	S-ZEST, without spatial correlations	ZEST, without spatial correlations	<0.001	0.127	<0.001
	ZEST, with spatial correlations	ZEST, without spatial correlations	<0.001	0.133	<0.001

These comparisons were calculated by using the average result of the simulation for each eye, so that the amount of simulations did not affect the significance of the *P* values. All comparisons were corrected for multiple discoveries using the Bonferroni–Holm method. The strategy on the left is generally the better performing of the two, with the exception of the MS-AE for the S-ZEST with spatial correlations in the presence of high FPs.

## Discussion

In our work, we demonstrate that DL structure–function predictions can be incorporated into perimetric strategies to improve both speed and accuracy of the test. For our simulations, we used robust estimates of the true sensitivity by taking the median of test–retest series in a glaucoma cohort with a wide range of VF damage. We also improved the accuracy of our simulations by including global fluctuations in sensitivity, which were specific to each tested eye as observed from actual test–retest experiments.

This is the first work investigating this application of DL structure–function predictions to directly improve perimetry test results. However, the framework of our work has been largely laid out in previous publications^15^^–^^17^ and was, in fact, heavily based on tools available within the OPI environment. This increases the translational value of our work, because the OPI can be directly interfaced with many commercially available perimeters,^18^ which would facilitate deployment in both clinical and research contexts. Important elements of novelty are the use of structural information from individual patients to characterise spatial correlations between neighboring locations in the VF and the incorporation of global fluctuations in sensitivity in our simulation technique. The latter is particularly relevant to assess the effectiveness of structural information in improving the reliability of global metrics, such as MS or MD, the variability of which is mainly determined by such global fluctuations.^27^^,^^28^

In our experiments, the MAE was reduced by 15% to 18% with S-ZEST in reliable simulated patients. This is remarkably close to the 20% benchmark estimated to be necessary to provide clinical benefit in the detection of pointwise deterioration.^30^ This is important with regard to facilitating the tracking of localized progression in clinical practice, especially for locations near fixation. S-ZEST also seemed able to reduce the effect of FP or FN responses. This is evident by looking at the mean signed error of S-ZEST (Table 2), which was closer to zero for both the high FP and high FN simulations compared to ZEST. It should be noted that a much smaller improvement was obtained for MS (Tables 3, 4; Fig. 4), and this is likely the case also for other global metrics, such as the MD, because correlated errors will determine global shifts in MS and dominate variability (see Appendix).^26^^,^^27^^,^^31^ Such a result is important to understanding what improvements should be expected in global metrics when new strategies are deployed in clinical practice. Naturally, our results depend on the specific choice of the variability model used to simulate the data. We adopted the exponential model for the SD of the Gaussian psychometric function proposed by Henson et al.^3^ capped at 6 dB. This choice was mainly done for consistency with previous work but does not have a strong justification. For example, in previous work, we found that a cap at 8.17 dB would better describe variability in a test–retest dataset.^32^ Gardiner et al.^33^ recently proposed alternative models on other psychometric data. They showed, for example, that a segmented linear model for the SD might better describe variability for extremely low threshold values. To show how our results are affected by the choice of the model, we performed additional simulations with reliable observers using the coefficients provided by Gardiner et al.^33^ Implementing a segmented linear model is not easily achieved in the current OPI simulation framework. However, the exponential model fitted on the same data and capped at 10 dB offers a very close approximation for thresholds above 0 dB,^33^ the lower bound for our data, and was therefore chosen for these additional experiments. These results are largely similar to our main ones, although they further favor S-ZEST, showing a significant effect on the MS-AE, which was not statistically significant in our main set of simulations (see Table 4). These additional results are reported as Supplementary Material.

The improvement in accuracy is greater than reported in previous similar work.^16^ Although caution should be used when comparing results from different datasets, it is reasonable to assume that most of the improvement is a result of the increased accuracy of the DL predictions compared to linear models.^9^^,^^15^^,^^16^ Such a theoretical improvement was predicted by Denniss et al.^17^ in their simulation work. S-ZEST was also considerably faster than traditional ZEST. Spatial correlations had a bigger effect on speed than on accuracy (Table 3, Fig. 4). It is interesting to note, however, that the use of DL structural predictions alone outperformed the use of spatial correlations in a standard ZEST in reducing the number of presentations (Fig. 4; Tables 3, 4). Interindividual variability in structure–function mapping can also affect the accuracy of the predictions, especially near the horizontal midline (corresponding to the anatomical raphe).^7^^,^^34^ We provide, as Supplementary Material, a plot of the MAE for each location in the VF. S-ZEST outperformed ZEST at all locations, including those where nasal step defects would be located.

Our DL model makes use of circumpapillary scans and gathers information from both reflectivity values and segmented RNFL thickness.^9^ Other DL models have combined multiple sources of information to derive VF estimates. For example, Kihara et al.^10^ combined optic disk photographs and SD-OCT scans through a policy network. Park et al.^13^ combined segmented thickness maps from macula and optic disk OCT scans to predict VF defects. However, somewhat useful VF predictions were also obtained from RNFL thickness values alone.^11^^,^^12^ All of these predictions have the potential to be used in this framework to improve VF testing, with the degree of improvement being related to the accuracy of the DL model. Naturally, algorithms that predict sensitivity values would be more advantageous compared to others designed to provide derived metrics, such as deviation from normative values.^11^

Additional improvements could be obtained by modeling longitudinal data to determine an individualized structure–function relationship for a specific eye or subject. This would be ideally achieved with a bespoke DL model, but it could also be obtained by modeling the conditional distribution of the measured sensitivity over time given the DL predictions of a cross-sectional model, such as the one used for our experiment. This characterization would also allow detecting global fluctuations in performance at the beginning of the test—for example, by testing seeding points in a growth pattern for the VF testing strategy^24^ to assess large deviation from an expected “best estimate” of the true sensitivity. This cannot be achieved if only individual tests are considered, because it is not possible to distinguish large differences between measurements and predictions due to noise or intersubject variability.

Obvious limitations pertain to the detection of VF defects not caused by glaucoma. For example, estimation of macular damage from geographic atrophy would be biased in patients with a relatively healthy ONH, possibly compromising the detection of a defect. Algorithms to predict VF sensitivity in macular pathology from OCT scans have been developed,^35^^,^^36^ but they focus on specific diseases, and a framework for the integration of different predictions has not yet been defined. It is likely that further advances in DL technology will generate models able to predict VF sensitivity taking into account multiple ocular diseases. However, VF loss due to extraocular reasons, such as strokes, might still suffer from underestimation. A potential solution could be to assess discordance between structural predictions and previous functional tests,^37^ to decide whether structural predictions could negatively affect the accuracy of the test. Additional information could come from developing models to predict the VF of both eyes from the same patient. Such models could exploit structural similarities (and differences) between the two eyes to improve their accuracy or detect binocular congruent changes caused by lesions to the optic chiasm or optic tract. Another technological limitation is the fact that most of these algorithms are designed to output sensitivity as numerical vectors of fixed size, such as the 52 locations of the 24-2 grid in our case. This limits their application to dynamic and customized grids, which might be needed to better characterize focal visual field loss (for example, in the macula^38^^–^^40^) or to improve the resolution at the edges of the scotoma.^41^

Our results are derived from test simulations. Despite our best effort to replicate realistic behavior, such as by including personalized profiles of global fluctuations, these results will require confirmation in real patients, and this will be the objective of future work.

There is a broader consideration to be made regarding the deployment of such strategies in clinical practice. Although improving structure–function concordance is generally a desirable outcome, especially in research, clinicians often use the disagreement between the two measurements to derive useful clinical information. For example, they might confirm progression detected with functional data with an independently measured structural change. Disagreement between RNFL and VF change can also be used to detect VF changes from diseases other than glaucoma, such as macular degeneration or neurological lesions. A more widely applicable implementation of the S-ZEST should allow ample freedom for these useful disagreements to occur, such as building structurally informed prior distributions from a large clinical dataset that would include patients with defects from glaucoma and other diseases.

## Supplementary Material

Supplement 1

## Figures and Tables

**Figure A1.**
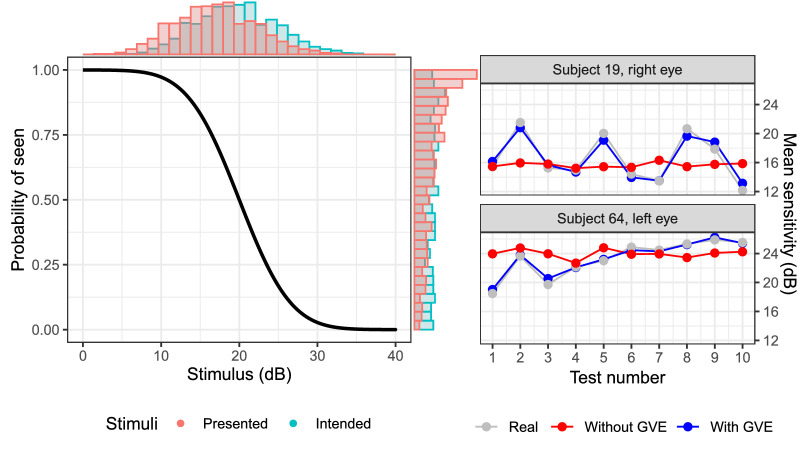
The *left panel* shows how the response from the psychometric function is sampled to include the global visit effect (GVE; positive shift in this example). The stimuli are biased toward brighter intensities (*red horizontal histogram*). This in turn increases the likelihood of positive responses (*red vertical histogram*). This can be compared with the responses obtained without bias (*teal histograms*). The bias for each location is part of a template that preserves the correlation across the visual field for each test. The *right panel* shows two examples in which neglecting the GVE leads to a severe underestimation of the fluctuations in mean sensitivity when compared to real data.

## Appendix

To account for global fluctuations in our simulations we adopted a method similar to Wu and Medeiros.^27^ We first transformed all of the observed sensitivities into deviations from their respective BAE sensitivity. Each one of these deviation values was transformed into a probability value through the psychometric function used for the simulations: a Gaussian cumulative distribution function centered on the BAE and with a standard deviation linked to the mean as in Henson et al.^3^ (see Methods). These probability values, generated for each location in each test, constituted a test *template* and capture the correlated fluctuations in test performance scaled according to the expected variability at each sensitivity. To apply the fluctuations in our simulations, a random template among the ones generated for a given eye was chosen at each iteration and applied to the BAE value. This meant re-transforming the probability value into a sensitivity offset from the BAE sensitivity based on the expected psychometric function and subtracting this value from the stimulus. A negative offset indicated worse performance, and the stimulus was therefore shifted toward higher dB values (dimmer) to increase the chances of no response. The opposite was true for a positive offset. This is similar to the method used by Chong et al.^42^ to simulate artificial scotomas in healthy observers. Because the probability values can be reported on any psychometric function and can therefore adapt to any simulated sensitivity, for each simulated test the values within the template were randomly shuffled to increase the number of available templates. However, shuffling was not applied to locations with a BAE sensitivity equal to 0 dB, because the BAE for these locations might not correspond to the true median of the test–retest distribution (i.e., the “true” sensitivity was not observed). Locations where no response was recorded in any of the tests were set to have a true sensitivity of 0 dB and were not allowed to fluctuate with the template. The effect of neglecting global fluctuations can be observed in the examples reported in Figure A1.
