# Supplementary material for: Improving the Accuracy and Speed of Visual Field Testing in Glaucoma With Structural Information and Deep Learning
Source: Transl Vis Sci Technol. 2023 Oct 13;12(10):10. doi: 10.1167/tvst.12.10.10 (PMC10587851; doi:10.1167/tvst.12.10.10)
Supplement: Supplement 1 [file tvst-12-10-10_s001.pdf]

# Supplementary to: Improving the accuracy and speed of visual field testing in glaucoma with structural information and deep learning

## Simulations with alternative variability models

The tables report the results of simulations with reliable observers (rate of false positive and false negative mistakes of 5%) using the exponential model for the standard deviation (SD) of the Gaussian psychometric function proposed by Gardiner et al.[1] capped at 10 dB ($SD_{dB} = 9.658 - 0.287 \times sensitivity_{dB}$).

| | Mean signed error | | Mean absolute error | | Average presentations | |
|---|---|---|---|---|---|---|
| | **ZEST** | **S-ZEST** | **ZEST** | **S-ZEST** | **ZEST** | **S-ZEST** |
| **w/o Spatial correlations** | 0.38 (3.91) | -0.16 (3.31) | 2.57 (2.98) | 2.07 (2.59) | 4.93 (2.33) | 4.11 (1.80) |
| **w/ Spatial correlations** | 0.26 (3.52) | -0.06 (3.12) | 2.28 (2.69) | 1.92 (2.46) | 4.77 (2.21) | 3.56 (2.06) |

| | MAE per eye | | MS-AE per eye | | Total presentations per test | |
|---|---|---|---|---|---|---|
| | **ZEST** | **S-ZEST** | **ZEST** | **S-ZEST** | **ZEST** | **S-ZEST** |
| **w/o Spatial correlations** | 2.57 (0.89) | 2.07 (0.70) | 0.50 (0.43) | 0.38 (0.26) | 256.39 (50.78) | 213.61 (27.41) |
| **w/ Spatial correlations** | 2.28 (0.79) | 1.92 (0.68) | 0.33 (0.28) | 0.25 (0.21) | 248.12 (47.62) | 185.11 (36.33) |

| Comparison | | p-values | | |
|---|---|---|---|---|
| | | **MAE** | **MS-AE** | **Total presentations** |
| **S-ZEST, w/ Spatial correlations** | **S-ZEST, w/o Spatial correlations** | <0.001 | <0.001 | <0.001 |
| **S-ZEST, w/ Spatial correlations** | **ZEST, w/ Spatial correlations** | <0.001 | 0.003 | <0.001 |
| **S-ZEST, w/ Spatial correlations** | **ZEST, w/o Spatial correlations** | <0.001 | <0.001 | <0.001 |
| **S-ZEST, w/o Spatial correlations** | **ZEST, w/ Spatial correlations** | <0.001 | 0.138 | <0.001 |
| **S-ZEST, w/o Spatial correlations** | **ZEST, w/o Spatial correlations** | <0.001 | <0.001 | <0.001 |
| **ZEST, w/ Spatial correlations** | **ZEST, w/o Spatial correlations** | <0.001 | <0.001 | <0.001 |

## Mean absolute error by location

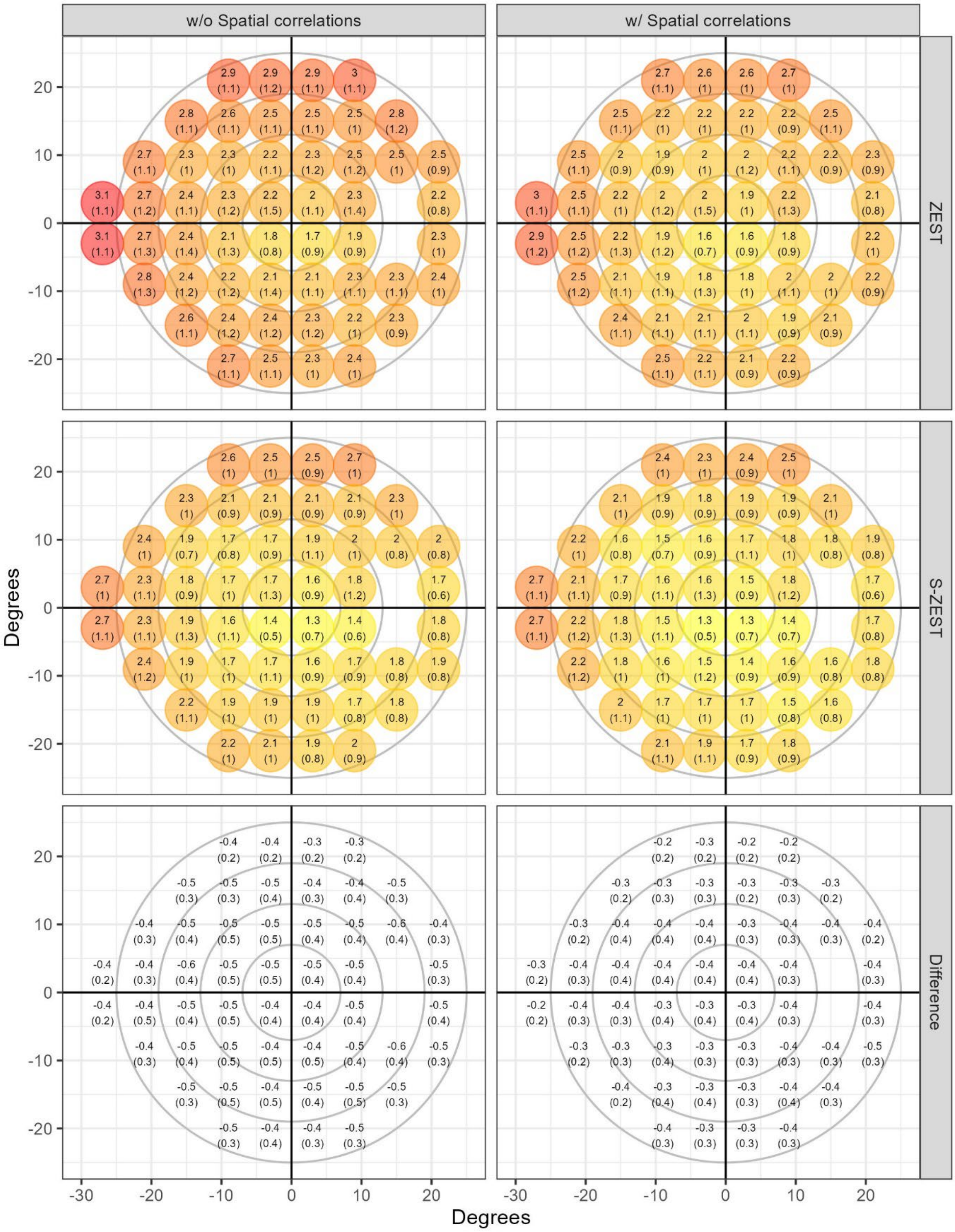


Absolute error (AE) at each location of the 24-2 grid, reported as Mean (across-eye Standard deviation). The colour gradient is proportional to the mean AE. The bottom row reports the per-eye mean difference (standard deviation) between S-ZEST and ZEST at AE at each location. The grey circles are visual guides to compare the mean AE for locations at approximately the same eccentricity at various distances from the horizontal midline, where inaccurate structure-function mapping of the location of the anatomical raphe might affect the results obtained with S-ZEST.

# References

1. Gardiner SK, Swanson WH, Mansberger SL. Long- and Short-Term Variability of Perimetry in Glaucoma. *Transl Vis Sci Technol* 2022;11:3.
